# PPARγ Transcription Deficiency Exacerbates High-Fat Diet-Induced Adipocyte Hypertrophy and Insulin Resistance in Mice

**DOI:** 10.3389/fphar.2020.01285

**Published:** 2020-08-19

**Authors:** Fusheng Guo, Shuangshuang Xu, Yanlin Zhu, Xing Zheng, Yi Lu, Jui Tu, Ying He, Lihua Jin, Yong Li

**Affiliations:** ^1^ State Key Laboratory of Cellular Stress Biology, Innovation Center for Cell Signaling Network, School of Life Sciences, Xiamen University, Xiamen, China; ^2^ Department of Diabetes Complications and Metabolism, Diabetes and Metabolism Research Institute, Beckman Research Institute, City of Hope National Medical Center, Duarte, CA, United States; ^3^ Laboratory Animal Center, Xiamen University, Xiamen, China

**Keywords:** PPARγ, DNA binding, transcriptional activity, metabolic disorder, mutant, obesity, insulin resistance

## Abstract

**Background:**

The transcriptional factor peroxisome proliferator–activated receptor γ (PPARγ) is an important therapeutic target for the treatment of type 2 diabetes. However, the role of the PPARγ transcriptional activity remains ambiguous in its metabolic regulation.

**Methods:**

Based on the crystal structure of PPARγ bound with the DNA target of PPARγ response element (PPRE), Arg134, Arg135, and Arg138, three crucial DNA binding sites for PPARγ, were mutated to alanine (3RA), respectively. *In vitro* AlphaScreen assay and cell-based reporter assay validated that PPARγ 3RA mutant cannot bind with PPRE and lost transcriptional activity, while can still bind ligand (rosiglitazone) and cofactors (SRC1, SRC2, and NCoR). By using CRISPR/Cas9, we created mice that were heterozygous for PPARγ-3RA (PPARγ^3RA/+^). The phenotypes of chow diet and high-fat diet fed PPARγ^3RA/+^ mice were investigated, and the molecular mechanism were analyzed by assessing the PPARγ transcriptional activity.

**Results:**

Homozygous PPARγ-3RA mutant mice are embryonically lethal. The mRNA levels of PPARγ target genes were significantly decreased in PPARγ^3RA/+^ mice. PPARγ^3RA/+^ mice showed more severe adipocyte hypertrophy, insulin resistance, and hepatic steatosis than wild type mice when fed with high-fat diet. These phenotypes were ameliorated after the transcription activity of PPARγ was restored by rosiglitazone, a PPARγ agonist.

**Conclusion:**

The current report presents a novel mouse model for investigating the role of PPARγ transcription in physiological functions. The data demonstrate that the transcriptional activity plays an indispensable role for PPARγ in metabolic regulation.

## Introduction

Obesity is a growing worldwide risk factor for many complications in health, such as type 2 diabetes (T2D) and non-alcoholic fatty liver disease (NAFLD) (Wild et al., 2004; Golay and Ybarra, 2005; Esser et al., 2014; Byrne and Targher, 2015). Peroxisome proliferator-activated receptors (PPARs) are a family of ligand-activated transcription factors that belong to the nuclear hormone receptor superfamily. There are two isoforms of PPARγ, γ1, and γ2. Both of the isoforms are transcribed from the same gene under the control of different promoters leading to a longer N-terminus in PPARγ2 (Fajas et al., 1997) (**Supplementary Figure 1**). PPARγ1 is expressed in various tissues and highly enriched in adipose tissues, while the expression of PPARγ2 is restricted to adipose tissues (Tontonoz et al., 1994). PPARγ is enriched in both white adipose tissue (WAT) and brown adipose tissue (BAT) (Tyagi et al., 2011). Like many nuclear receptors, PPARγ contains five functional regions: an N-terminal activation function-1 (AF-1) domain (A/B domain), a DNA-binding domain (DBD, domain C), a ligand-binding domain (LBD, domain E), a hinge region that links the DBD and the LBD (domain D), and a C-terminal AF-2 domain in LBD (Jin and Li, 2010). Upon the binding with ligands by its LBD, PPARγ locates to the specific PPAR response element (PPRE) *via* its DBD as a heterodimer with retinoid X receptor (RXR), and recruits cofactors to regulate the transcription of many direct downstream target genes (Larsen et al., 2003; Jin and Li, 2010; Seale, 2010; Ahmadian et al., 2013). These target genes include glucose transporter type 4 (Glut4), phosphoenolpyruvate carboxykinase (PEPCK) (Tontonoz et al., 1995), fatty acid translocase (FAT/CD36) (Teboul et al., 2001), aquaporin 7 (AQP7, also named AQPap) (Kishida et al., 2001), adipocyte fatty acid binding protein (aP2) gene (Rival et al., 2004), stearoyl-CoA desaturase 1 (SCD1) (Miller and Ntambi, 1996), and uncoupling protein 1 (UCP1) (Petrovic et al., 2010), etc., that are involved in a variety of processes including adipocyte differentiation, glucose metabolism, and insulin sensitivity (Larsen et al., 2003; Jin and Li, 2010; Seale, 2010; Ahmadian et al., 2013). Therefore, PPARγ has been a primary pharmacological target for drug discovery for the treatment of obesity and T2D.

Rosiglitazone (Avandia) and pioglitazone (Actos) belong to an anti-diabetic drug class that targets PPARγ, called thiazolidiniones (TZDs) (Larsen et al., 2003; Karak et al., 2013). As full agonists of PPARγ, TZDs induce the transcription and expression of hundreds of genes by activating PPARγ. Some of these activated genes enhance insulin sensitivity, leading to the therapeutic effects; while activation of some other genes are thought to be the causes of adverse effects of TZDs including weight gain, fluid retention, congestive heart failure, and bone fractures (Ahmadian et al., 2013; Wright et al., 2014). These adverse effects caused by PPARγ full agonists might override the glycemic benefits in T2D patients. In fact, rosiglitazone has ever been suspended by the European Medicines Agency and restricted by the U.S. FDA.

The concept of a disconnect between the agonism potency of PPARγ agonists and their therapeutic property has been proposed years ago (Jones, 2010). Much evidence show that partial agonists of PPARγ, also called selective PPARγ modulators (SPPARM) with poor agonist activities, such as MRL24, INT-131, and MBX-102, exert good anti-diabetic property with fewer adverse effects (Acton et al., 2005; Gregoire et al., 2009; Taygerly et al., 2013). Actually, ligands that do not possess transcriptional agonism can potentially exhibit anti-diabetic property with little adverse effect by blocking Cdk5-mediated phosphorylation of PPARγ (Choi et al., 2011). Therefore, the characterization of PPARγ transcriptional activity in drug discovery remains unclear till now.

The controversy over TZD drugs as diabetic treatment has weakened confidence in developing drugs that target the PPAR family of nuclear receptors. Reports have demonstrated that heterozygous PPARγ-deficient mice exhibit improved insulin sensitivity (Kubota et al., 1999; Miles et al., 2000), supporting the negative role of PPARγ. However, there are also other reports suggesting that PPARγ bearing mutations in DBD or LBD are associated with lipodystrophy (Barroso et al., 1999; Freedman et al., 2005; Agostini et al., 2006; Jeninga et al., 2007). Therefore, there is a dire need to create an applicable model to clarify the role of the transcriptional activity of PPARγ in metabolism. Based on the crystal structure of the PPARγ–RXRα complex bound to PPRE (Chandra et al., 2008), we identified three crucial residues (Arg134, Arg135, and Arg138) on PPARγ that control the binding ability of PPARγ with PPRE and the subsequent transcriptional activity of PPARγ. Therefore, we created a transgenic mouse model containing the three point mutations (R134/135/138A, 3RA) to study the role of PPARγ transcription in metabolism.

## Materials and Methods

### Protein Purification

Human PPARγ containing domains from DBD to the C-terminus (CDE domains, residues 103–477) was expressed as an N-terminal 6×His fusion protein (H6-PPARγ CDE) from the expression vector pET24a (Novagen, Germany). 3RA mutant plasmid was constructed by site-directed mutagenesis with forward primer: AGGATGCAAGGGTTTCTTCGCGGCAACAA

TCGCATTGAAGCTTATCTATGACAG, and reverse primer: CTGTCATAGATAAGCTTCA

ATGCGATTGTTGCCGCGAAGAAACCCTTGCATCCT, using Pfu DNA polymerase (Thermo Fisher Scientific, USA). BL21(DE3) cells transformed with the expression plasmids were grown in LB broth at 25°C to an OD_600_ of approximately 1.0 and induced with 0.1 mmol/L isopropyl 1-thio-β-d-galactopyranoside (IPTG) at 16°C. Cells were harvested and sonicated in 100 ml of extract buffer (20 mmol/L Tris pH8.0, 150 mmol/L NaCl, 10% glycerol, and 25 mmol/L imidazole) per 2 liters of cells. After sonication, the lysate was centrifuged at 20,000 rpm for 30 min, and the supernatant was loaded on a 5-ml NiSO_4_-loaded HiTrap HP column (GE Healthcare, PA, USA). The column was washed with extract buffer, and the protein was eluted with a gradient of 25 to 500 mmol/L imidazole. The PPARγ CDE was further purified with a SP-Sepharose column (GE Healthcare, PA, USA).

### AlphaScreen Assay

The binding of H6-PPARγ CDE wild-type (WT) or H6-PPARγ CDE 3RA mutant protein with biotin-labeled PPRE was determined by AlphaScreen assay using a hexahistidine detection kit from Perkin-Elmer. PPRE was prepared by annealing biotin-PPRE-F: AGGGGACCAGGACAAAGGTCACGTTCGGGA and biotin-PPRE-R: TCCCGAACGTGACCTTTGTCCTGGTCCCCT, both of which with 5’ end biotin-labeled. The assay was performed in a buffer containing 50 mmol/L MOPS, 50 mmol/L NaF, 0.05 mmol/L CHAPS, and 0.1 mg/ml bovine serum albumin, all adjusted to a pH of 7.4. The binding assay was performed with 100 nM of protein with gradient doses of biotin-PPRE, or 1 nM of biotin-PPRE with gradient doses of H6-PPARγ CDE with or without 1 µM of rosiglitazone. For the binding of PPARγ CDE with cofactors peptide motifs in response to rosiglitazone, AlphaScreen assay was performed with 100 nM of PPARγ CDE, 100 nM biotin-labeled peptides with gradient doses of rosiglitazone. The sequences of the peptides: SRC1-2, SPSSHSSLTERHKILHRLLQEGSP; SRC2–3, QEPVSPKKKENALLRYLLDKDDTKD; and NCoR-2, GHSFADPASNLGLEDIIRKALMGSF.

### Dual Luciferase Report Assay

HEK-293T cells (ATCC, USA) were maintained in DMEM containing 10% fetal bovine serum (FBS) and were transiently transfected using Lipofectamine 2000 reagent (Thermo Fisher Scientific, USA). 24-well plates were plated 24 h prior to transfection (5 x 10^4^ cells per well). 200 ng of pcDNA3.1-Flag-PPARγ WT or 3RA mutant plasmid was co-transfected with 200 ng of PPRE-luc reporter plasmid into cells (Zheng et al., 2013). Renilla was co-transfected as an internal control. 1 µM of rosiglitazone or DMSO was added 5 h after transfection. Cells were harvested 24 h later for the luciferase assays. Luciferase activities were analyzed as the instruction of CheckMate™ Mammalian Two-Hybrid System (Promega, USA).

### Generation of PPARγ^3RA/+^ Mice

A PPARγ BAC clone was screened and isolated from BAC library, mapped by restriction digests and sequenced. The arginine 134, 135, and 138 residues in exon 5 were all paralleled mutated to alanine (3RA) using overlap PCR, and the fragment was cloned into a targeting vector that contains exon 5 homology arm. Meanwhile, the vector contains cassette with a floxed pGK-neo^r^. The vector was then delivered to embryonic stem (ES) cells (C57BL/6) *via* electroporation, followed by G418 selection, PCR screening, and Southern blot confirmation. Targeted lines were expanded and electroporated with a Cre recombinants expression vector to delete the neo^r^-cassette. Some correct targeted ES clones were selected for blastocyst microinjection, followed by chimera production in C57BL6 background. These mice were then interbred to obtain different genotypes littermate mice for experiments. Mice were maintained under environmentally controlled conditions with free access to diet and water. Animal experiments were conducted in the barrier facility of the Laboratory Animal Center, Xiamen University, approved by the Institutional Animal Use and Care Committee of Xiamen University, China. The methods were carried out in accordance with the approved guidelines.

### Mice Treatment

8 week-old male PPARγ^3RA/+^ and WT littermates were fed with a high-fat diet (HFD, 60% kcal fat, D12492, Research Diets Inc, USA). The body weight of mice were weighed weekly and the food intake was assessed every 4 weeks. Blood samples were obtained by the tail-cut method for small samples every 4 weeks for detecting blood glucose and insulin levels. After 15 weeks of HFD, mice were euthanized after 6 h of fasting. For rosiglitazone treatment study, mice were divided into two groups after a 15-week HFD, and intraperitoneally (i.p.) injected once daily with vehicle (40% of 2-hydroxypropyl-β-cyclodextrin, HBC, Sigma, USA) or 3 mg/kg of rosiglitazone for 6 days. Mice were euthanized after 6 h of fasting. For all mice research, part of liver and fat tissues was fixed in 4% paraformaldehyde for hematoxylin and eosin (H&E) staining by standard procedures. Other tissues were collected and frozen in liquid nitrogen for use. Serum was collected for the measurement of metabolic parameters. Animal experiments were conducted in the barrier facility of the Laboratory Animal Center, Xiamen University, approved by the Institutional Animal Use and Care Committee of Xiamen University, China.

### Metabolic Parameters

Serum glucose level was analyzed using glucose oxidase method (Applygen, Beijing, China) (Wang C. et al., 2016). Serum insulin level was determined by ELISA using an ultra-sensitive mouse insulin kit (Crystal Chem, USA) (Ding et al., 2016). Serum levels of total cholesterol, triglycerides, LDL-C, HDL-C, and free fatty acids (FFA) levels were assayed using the calorimetric kits from Nanjing Jiancheng Bioengineering Institute (Nanjing, China) (Jiang et al., 2016; Wang et al., 2019; Liu et al., 2020). Liver TG was analyzed using Tissue triglyceride assay kit (Applygen, Beijing, China) (Wang C. et al., 2016).

### GTT and ITT

Glucose tolerance test (GTT) and insulin tolerance test (ITT) were performed in mice before and after a 15-week HFD feeding. For the GTT, mice were fasted for 16 h with free access to water, and then orally gavaged with 1 g/kg body weight of glucose. Blood glucose level was assessed with the Accu-Check Performa (Roche Applied Science, Mannheim, Germany) at 0, 15, 30, 60, 90, and 120 min. For the ITT, mice were fasted for 6 h with free access to water, and then i.p. injected with 1 U/kg of recombinant human insulin (Novolin 30R; Novo Nordisk, Bagsvaerd, Denmark). Blood glucose level was measured at 0, 15, 30, 60, and 120 min after insulin injection.

### Gene Expression

The protein level of PPARγ in inguinal WAT (iWAT) was assessed by western blot using mouse monoclonal anti- PPARγ (Santa Cruz, Cat. No. sc-7273, 1:1000) (Chakraborty et al., 2019; Jung et al., 2019) and mouse monoclonal anti-β-actin (Protein Tech, Cat. No. 60008-1-Ig, 1:2000). Total RNA was isolated from liver and fat tissues using Tissue RNA kit (Omega Bio-Tek, GA). The first strand cDNA was reverse-transcribed using TAKARA reverse transcription kit. Real-time quantitative PCR reactions were performed with SYBR Premix Ex TaqTM (TAKARA) on a CFX96™ Real-Time PCR Detection System (Bio-Rad). Relative mRNA expression levels were normalized to β-actin levels. The sequences of the primers used were listed in **Supplementary Table 1**.

### Statistical Analysis

Values were expressed as mean ± standard error of mean (SEM). Statistical differences were calculated by one-way ANOVA followed by the Dunn’s test or Student’s *t* test. Statistical significance was shown as *p<0.05, **p<0.01 or ***p<0.001.

## Result

### R134/135/138A Mutations Abolish the PPRE-Binding Ability and the Transcriptional Activity of PPARγ

**Figure 1:**
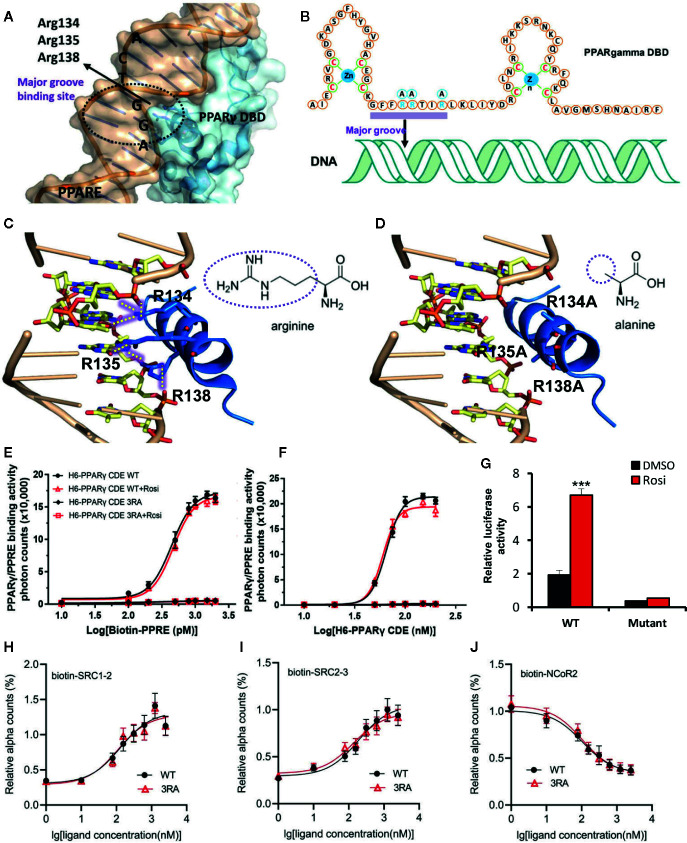
R134/135/138A mutations abolish the PPRE-binding ability and the transcriptional activity of PPARγ. **(A)** The structure of PPRE and PPARγ interaction shows that Arg134, Arg135 and Arg138 locate into the major groove of PPRE. PPRE is colored in light orange, and PPARγ DBD is in sky blue. **(B)** Binding model of PPARγ DBD on PPRE. Zinc finger domains of PPARγ DBD and Arg134, Arg135 and Arg138 are shown in the sequence of PPARγ DBD. **(C**, **D)** The binding of wild type (WT) PPARγ with PPRE **(C)** and the predicted binding model of R134/135/138A (3RA) mutant PPARγ with PPRE **(D)**. Hydrogen bonds are indicated by dotted yellow lines. PPRE is colored in light orange, and the helix 1 of PPARγ DBD is in blue. The different groups of arginine and alanine are shown. The structure images in **Figures 1A, C, D** were generated by open source software PyMOL 099rc6 (www.pymol.org) and the chemical structures were drawn by chemdraw2014. **(E)** Dose response curve of biotin-PPRE with 100 nM of WT or 3RA mutant PPARγ CDE by AlphaScreen assay. **(F)** Dose response curve of WT or 3RA mutant PPARγ CDE with 1 nM of biotin-PPRE by AlphaScreen assay. Rosiglitazone (1 μM) does not affect the binding affinity of both WT and 3RA mutant PPARγ in **(E**, **F)**. **(G)** Transcriptional activity of WT or 3RA mutant PPARγ by rosiglitazone. HEK-293T cells were co-transfected with WT or 3RA mutant pcDNA3.1-Flag-PPARγ plasmid together with PPRE-luc reporter plasmid. Renilla was co-transfected as an internal control. 1 µM of rosiglitazone or DMSO was added 5 h after transfection. Cells were harvested 24 h later for the luciferase assays. **(H**–**J)** 3RA mutation does not affect the interaction of PPARγ CDE with rosiglitazone and co-factors. Dose curves of the interaction between PPARγ CDE WT/3RA and biotin-labeled cofactors: coactivator peptides SRC1-2 **(H)** and SRC2-3 **(I)**, and corepressor peptide NCoR2 **(J)**, in response to rosiglitazone by AlphaScreen assay. For **(E–J)**, experiments were performed in triplicate and repeated three times with similar results. Data show a representative experiment. Values are means ± SEM, ***p < 0.001 by one-way ANOVA followed by the Dunn’s test.

To evaluate the role of the transcriptional function of PPARγ on metabolism, we attempted to create a mouse model that is deficient in the transcriptional activity of PPARγ while the DBD-independent actions of PPARγ are intact. Because PPARγ needs to bind to PPRE to activate the downstream transcription, we searched for crucial sites in PPARγ to destroy its binding on PPRE. Based on the crystal structure of the PPARγ–RXRα complex bound to PPRE (Chandra et al., 2008), we found that the Arg134, Arg135, and Arg138 residues in PPARγ form six hydrogen bonds in the major groove of the PPRE double helix (**Figures 1A–C**). However, if the three arginine residues were mutated into alanine, the six hydrogen bonds will not form (**Figure 1D**). The absence of the hydrogen bonds is predicted to abolish the binding between PPARγ and PPRE while sparing other functions crucial for the transcriptional activity of PPARγ including the zinc finger structure of PPARγ DBD and the ligand-binding LBD (Chandra et al., 2008).

Therefore, we mutated the three arginine residues at Arg134, Arg135, and Arg138 of PPARγ to alanine (named PPARγ-3RA). The binding ability of PPAR-3RA with PPRE was studied by using AlphaScreen assay. 6 × His tag- PPARγ WT or PPARγ-3RA that contains the domains ranging from DBD, hinge, and the C-terminus LBD of PPARγ (H6-PPARγ CDE) was expressed in BL21 (DE3) and purified for the assay. As expected, the binding signal of the H6-PPARγ CDE WT increased in a PPRE concentration-dependent manner (**Figure 1E**). In contrast, H6-PPARγ CDE 3RA did not show any binding signal with increasing concentration of PPRE. We obtained similar results when a gradient concentration of H6-PPARγ CDE WT or H6-PPARγ CDE 3RA was used to bind PPRE (**Figure 1F**). Additionally, the same result was produced with the treatment of rosiglitazone, an agonist for PPARγ (**Figures 1E, F**), due to the ligand-independent nature of the binding ability of DBD domain to DNA, which is distinct from LBD (Rastinejad et al., 2013). These data confirmed that the PPARγ-3RA mutant lost the binding ability toward PPRE. Next, we tested the transcriptional activity of the PPARγ-3RA mutant. WT or 3RA mutant pcDNA3.1-Flag-PPARγ was co-transfected with PPRE-luc plasmid into HEK-293T cells for luciferase reporter assay. The result showed that the transcriptional activity of PPARγ dramatically decreased after 3RA mutation. Rosiglitazone treatment significantly induced the transcriptional activity of WT PPARγ, but failed to activate PPARγ-3RA (**Figure 1G**).

To test if the 3RA mutations affect the binding ability of PPARγ with ligands and cofactors, we performed an AlphaScreen assay using PPARγ CDE WT or 3RA protein and biotin-labeled cofactors peptides in response to rosiglitazone. The results showed that both WT and 3RA mutant of PPARγ CDE can recruit co-activators SRC1 and SRC2 and release co-repressor NCoR in response to rosiglitazone (**Figures 1H–J**). Our data demonstrate that although PPARγ 3RA mutant cannot bind with PPRE (**Figures 1E, F**), the mutant is still able to bind ligands and cofactors such as SRC1, SRC2, and NCoR.

Together, these results demonstrate that the PPARγ-3RA lost the binding ability toward PPRE and further the DBD-dependent ligand-regulated transcriptional activity of PPARγ, while maintains the ability to bind ligands such as rosiglitazone and cofactors, such as SRC1, SRC2, and NCoR.

### Decreased Transcriptional Ability of PPARγ in Heterozygous PPARγ^3RA/+^ Mice

**Figure 2:**
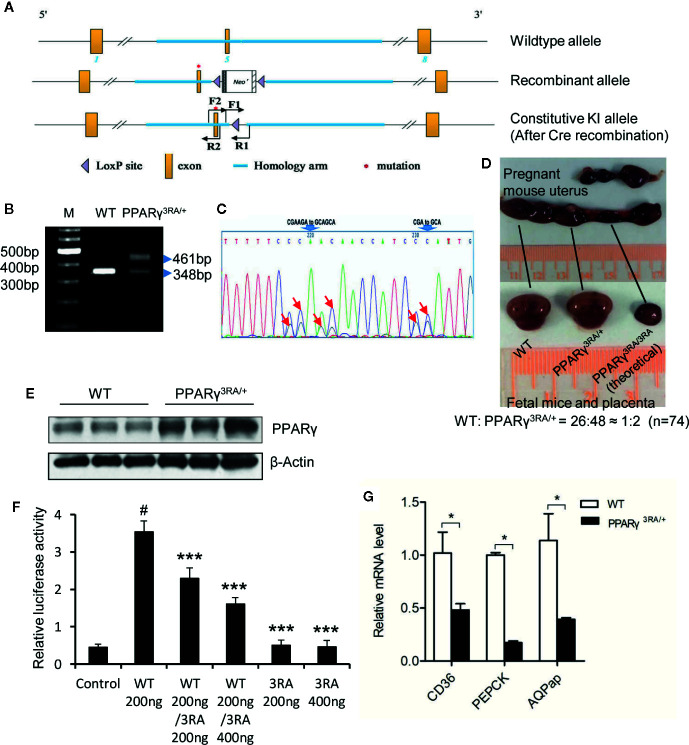
Generation of PPARγ 3RA mutant mouse model. **(A)** Schematics of the targeting strategy. Top: WT allele. Middle: recombinant allele. Bottom: constitutive knock-in allele after Cre recombination. **(B)** Genotyping. Genomic DNA extracted from mouse tail was used for PCR with primer pair F1/R1. The PCR product with one band of 348 bp represents the WT PPARγ, and PCR product with two bands (348 bp and 461 bp) represents the heterozygotes. **(C)** Sequencing analyses of PCR product using primer pair F2/R2 verified the mutations in PPARγ gene. Heterozygotes show two peaks in the codes of Arg134, Arg135 and Arg138, where one peak indicates the allele of wild-type (CGAAGA and CGA), the other is 3RA mutant (GCAGCA and GCA). The mutated nucleotides were indicated by arrows. **(D)** Homozygous PPARγ 3RA mutant (PPARγ^3RA/3RA^) mice are embryonic death. Heterozygous pregnant (mate with a heterozygous male) was dissected, about a quarter of the fetal mice in the womb were absorbed by the mother, leaving only the placenta. The ratio of newborn cubs (homozygote/heterozygote) is about 1/2 when we calculated total 74 cubs (n=74). **(E)** The protein level of PPARγ in the fat tissues of WT and PPARγ^3RA/+^ mice. **(F)** *In vitro* reporter assay. HEK-293T cells were co-transfected with indicated quantity of WT and 3RA mutant pcDNA3.1-Flag-PPARγ plasmid together with PPRE-luc reporter plasmid. Empty pcDNA3.1-Flag vector was the control. Renilla was co-transfected as an internal control. 1 µM of rosiglitazone was added 5 h after transfection. Cells were harvested 24 h later for the luciferase assays. Experiments were performed in triplicate and repeated three times with similar results. Data show a representative experiment. Values are means ± SEM, ^#^p<0.001 versus vector control, ***p < 0.001 versus WT 200 ng, one-way ANOVA followed by the Dunn’s test. **(G)** Relative mRNA level of PPARγ direct target genes in fat tissues of WT and PPARγ^3RA/+^ mice by quantitative RT-PCR. Experiments were repeated three times with similar results. Data show a representative experiment. Values are means ± SEM, n=6 per group. *p < 0.05 by Student’s t test.

We mutated codons for amino acids 134, 135, and 138 of the mouse PPARγ gene from CGA (arginine), AGA (arginine) and CGA (arginine) to GCA (alanine), respectively, *via* gene targeting in mouse ES cells and created genetically modified mouse carrying the 3RA mutations (**Figures 2A–C**). In 74 progenies born from PPARγ^3RA/+^ intercrosses, no PPARγ^3RA/3RA^ homozygous mice were obtained. WT and PPARγ^3RA/+^ littermates were born at the expected Mendelian ratio (26:48 ≈ 1:2) (**Figure 2D**), indicating that the PPARγ 3RA mutations cause embryonic lethality due to the loss of PPARγ transcriptional activity. These results suggest that the transcriptional function of PPARγ is essential for embryonic development.

To confirm the decreased transcriptional ability of PPARγ in heterozygous PPARγ^3RA/+^ mice, we analyzed gene expression in iWAT from WT and PPARγ^3RA/+^ mice. The expression level of total PPARγ was higher in iWAT from PPARγ^3RA/+^ than WT mice (**Figure 2E**) which might be the compensatory expression due to the loss of transcriptional activity for PPARγ 3RA mutation *in vivo*. Our *in vitro* reporter assay indicated that co-existence of PPARγ 3RA mutant significantly reduced the transcriptional activity of WT PPARγ (**Figure 2F**), further supporting the decreased transcriptional activity in heterozygous PPARγ^3RA/+^ mice. As expected, the mRNA levels of genes that are directly downstream of PPARγ, such as FAT/CD36, PEPCK, and AQPap, were significantly lower in iWAT of PPARγ^3RA/+^ mice compared to those of WT littermates (**Figure 2G**). The differential mRNA expression level of PPARγ downstream genes in WT and PPARγ^3RA/+^ mice confirmed the impaired transcriptional activity of PPARγ in the PPARγ^3RA/+^ mice.

### PPARγ 3RA Mutations in Mice Exacerbate HFD-Induced Obesity and Adipocyte Hypertrophy

**Figure 3:**
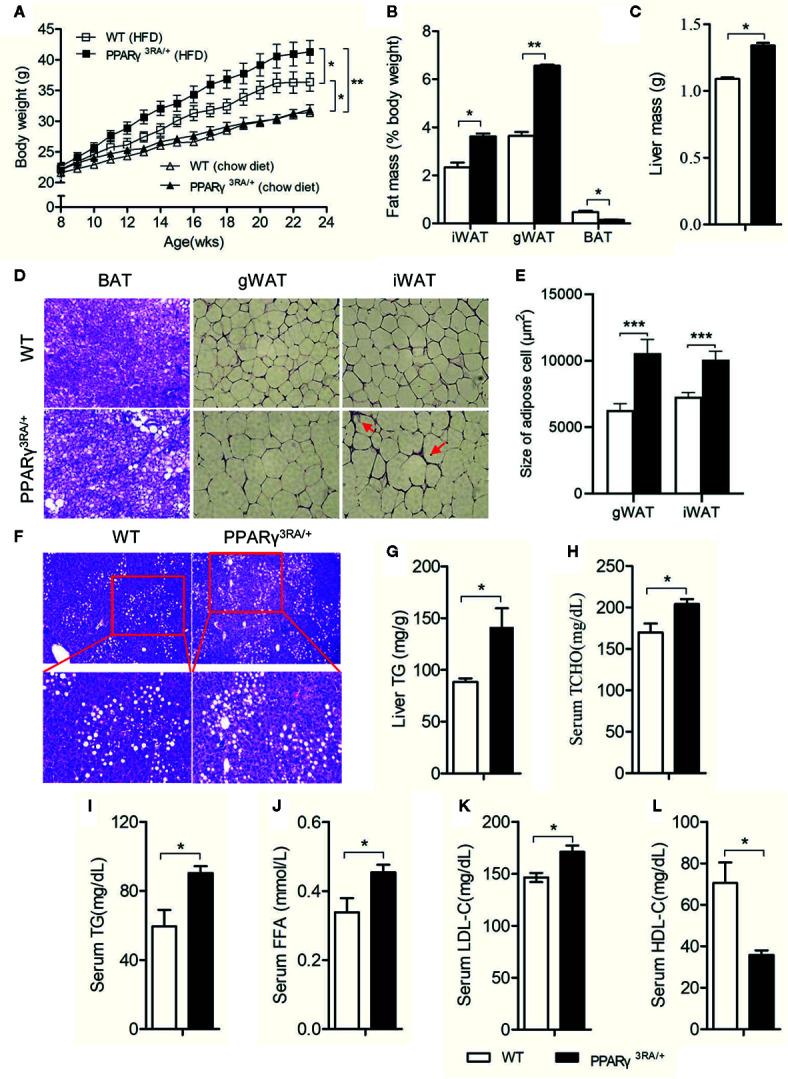
PPARγ 3RA mutations in mice exacerbate HFD-induced obesity and adipocyte hypertrophy. **(A)** Body weight of PPARγ^3RA/+^ and WT littermates during a 15 weeks HFD feeding or normal chow diet. Data from the final time point were compared. Samples were collected from 15-week HFD-fed mice for assays. **(B)** Weights of white adipose tissue (inguinal white fat, iWAT; gonadal white fat, gWAT), brown fat (BAT) and **(C)** liver mass from PPARγ^3RA/+^ and WT littermates. **(D)** Representative histological analysis of iWAT, gWAT and BAT from PPARγ^3RA/+^ and WT littermates under a HFD by H&E staining. Inflammatory infiltration was labeled by red arrows. Original magnification, 200×. **(E)** Quantitative statistics of adipocyte size in **(D)**. **(F)** Representative images of H&E stained liver sections of PPARγ^3RA/+^ and WT littermates. Original magnification: top, 100×; bottom, 200×. **(G)** Hepatic triglyceride level of PPARγ^3RA/+^ and WT littermates. **(H**, **I)** Fasting serum levels of total cholesterol (TCHO) **(H)**, triglycerides (TG) **(I)**, free fatty acids (FFA) **(J)**, LDL-C **(K)** and HDL-C **(L)** of PPARγ^3RA/+^ and WT littermates. Values are means ± SEM, n=12 per group, *p < 0.05, **p < 0.01 and ***p < 0.001 by Student’s *t* test. For **(G**–**L)**, measurement were repeated three times with similar results. Data show a representative experiment.

Under chow diet, the WT and PPARγ^3RA/+^ littermates showed similar phenotypes in body weight, liver/body weight ratio, fat/body weight ratio, as well as the histological analysis of the brown adipose tissue (BAT), WATs and the liver tissue (**Figure 3A** and **Supplementary Figure 2**). The similarity in these parameters suggests that the transcriptional activity of one PPARγ allele is enough for maintaining basic metabolism in the absence of external stimuli. Interestingly, when fed HFD, PPARγ^3RA/+^ mice gained significantly more body weight than WT mice did (**Figure 3A**) even though food intake was similar (**Supplementary Figure 3**). After 15 weeks of HFD feeding, the WATs and livers of PPARγ^3RA/+^ mice weighed significantly more than those of WT mice, while the BAT of PPARγ^3RA/+^ mice weighed significantly less than that of WT mice (**Figures 3B, C**). Histological analysis by H&E staining showed larger adipocytes size in the sections of iWAT, gonadal WAT (gWAT), and BAT from PPARγ^3RA/+^ mice than those of WT mice (**Figures 3D, E**). These results indicate that heterozygous PPARγ deficiency leads to more severe hypertrophy in white adipocytes and more whitening in brown adipocytes in mice under HFD. Notably, there was also visible inflammatory infiltration in iWAT of PPARγ^3RA/+^ mice (**Figure 3D**). Additionally, histological examination showed that heterozygous PPARγ deficiency leads to more lipid accumulation in the liver of mice under HFD (**Figure 3F**), which was further confirmed by the biochemical analysis of the hepatic triglycerides level (**Figure 3G**). As the results shown, the fasting plasma levels of total cholesterol (TCHO) (**Figure 3H**), triglyceride (TG) (**Figure 3I**), LDL-C, and FFA (**Figures 3J, K**) of PPARγ^3RA/+^ were all significantly higher than those of WT littermates, whereas the level of HDL-C (**Figure 3L**) was significantly lower. These results demonstrate that PPARγ 3RA mutations exacerbated HFD–induced obesity and adipocyte hypertrophy in mice.

### PPARγ 3RA Mutations in Mice Exacerbate HFD-Induced Insulin Resistance

**Figure 4:**
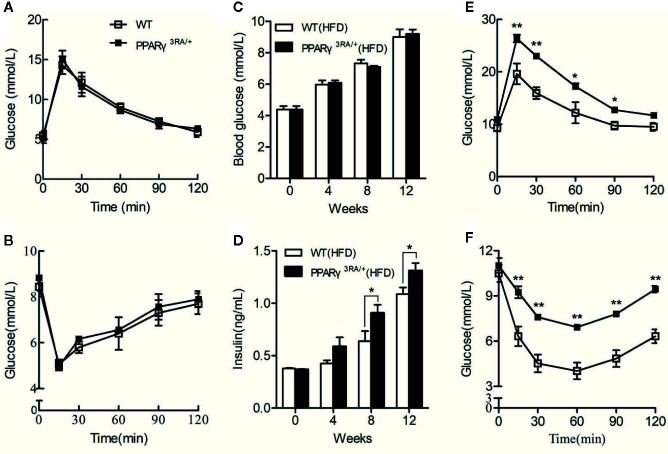
PPARγ 3RA mutations exacerbate HFD-induced insulin resistance in mice. Oral glucose tolerance test (OGTT) **(A)** and intraperitoneal insulin tolerance test (IPITT) **(B)** of 8-week old PPARγ^3RA/+^ and WT littermates with chow diet. From 8 weeks age, mice were fed with HFD for 15 weeks. Fasting blood glucose **(C)** and insulin **(D)** levels of mice fed with HFD. OGTT **(E)** and IPITT **(F)** of mice fed with HFD for 15 weeks. For **(C, D)**, values are means ± SEM, n=6 per group, *p < 0.05, **p < 0.01 by Student’s *t* test.

Chow diet-fed PPARγ^3RA/+^ and WT littermates showed similar fasting blood glucose level, glucose tolerance, and insulin tolerance (**Figures 4A, B**). When fed HFD, PPARγ^3RA/+^ and WT mice maintained similar fasting blood glucose levels (**Figure 4C**). However PPARγ^3RA/+^ mice showed significantly higher level of fasting blood insulin from 8 weeks after HFD-feeding (**Figure 4D**). Furthermore, PPARγ^3RA/+^ mice showed impaired glucose tolerance and insulin tolerance compared to their WT counterparts, suggesting that PPARγ 3RA mutations in mice exacerbate HFD-induced insulin resistance (**Figures 4E, F**). Taken together, our data demonstrate that the decreased transcriptional activity of PPARγ in PPARγ^3RA/+^ mice led to impairment of lipid and glucose metabolism under HFD.

### Metabolic Disorders in HFD-Fed PPARγ^3RA/+^ Mice Were Improved by Rosiglitazone Treatment

**Figure 5:**
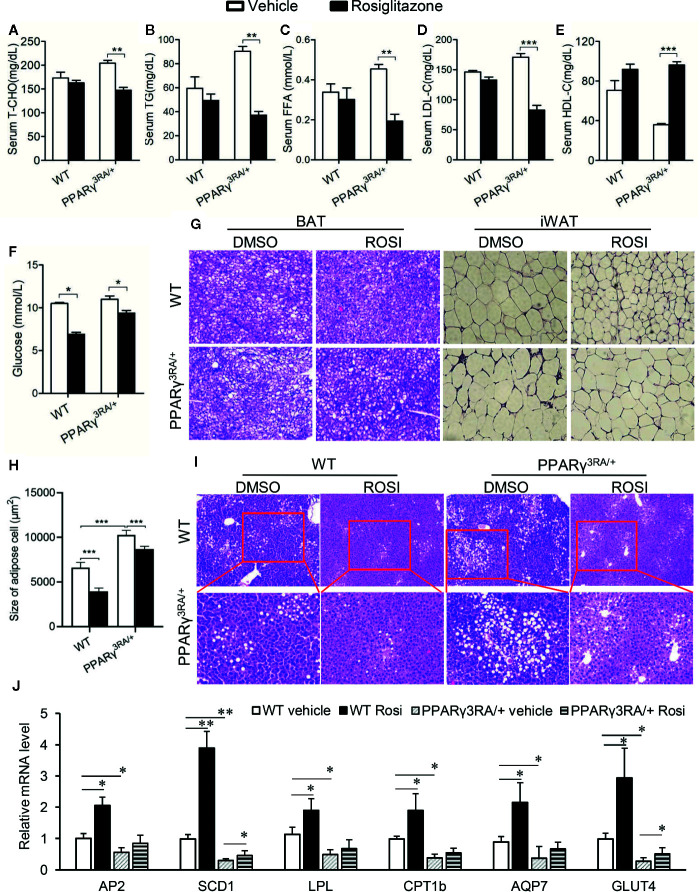
Metabolic disorders in HFD-fed PPARγ^3RA/+^ mice were improved by rosiglitazone treatment. 8-week old male PPARγ^3RA/+^ and WT littermates were fed with HFD for 15 weeks, and then i.p. injected with 3 mg/kg of rosiglitazone once daily for 6 days. **(A**–**F)** Fasting serum levels of TCHO **(A)**, TG **(B)** FFA **(C)**, LDL-C **(D)** HDL-C **(E)** and glucose **(F)**. **(G)** Representative images of H&E stained BAT and iWAT (original magnification, 200×). **(H)** Quantitative statistics of adipose cell size in **(G)**. **(I)** Representative images of H&E stained liver sections (original magnification: top, 100×; bottom, 200×). **(J)** mRNA levels of PPARγ direct target genes involved in glucose and lipid metabolism in liver tissues of mice. For **(A**–**F**, **J)**, measurement were repeated three times with similar results. Data show a representative experiment. Values are means ± SEM, n=6 per group, *p < 0.05, **p < 0.01 and ***p < 0.001 by one-way ANOVA followed by the Dunn’s test.

Next, we aimed to investigate whether or not the metabolic disorders induced by HFD in PPARγ^3RA/+^ mice could be reversed by increasing the transcriptional activity of PPARγ. Rosiglitazone was administered at 3 mg/kg once daily for 6 days to PPARγ^3RA/+^ and WT littermates that had been fed HFD for 15 weeks. Metabolic parameters were studied after the treatment. As shown in **Figure 5**, rosiglitazone treatment significantly reduced or showed the tendency to reduce the levels of cholesterol, triglyceride, FFA, LDL-C, and glucose in the serum, while increased the level of HDL-C in the serum of both PPARγ^3RA/+^ and WT littermates (**Figures 5A–F**). Histological examination further showed that after HFD feeding, both WT and PPARγ^3RA/+^ mice administrated with rosiglitazone showed less fat vacuoles in BAT and smaller adipocyte size in WAT (**Figures 5G, H**). Notably, rosiglitazone administration significantly improved the inflammation in WAT of HFD-fed PPARγ^3RA/+^ mice (**Figure 5G**). Additionally, rosiglitazone treatment not only efficaciously improved the hepatic steatosis in WT mice, but also in PPARγ^3RA/+^ mice (**Figure 5I**).

We further investigated the mRNA levels of PPARγ target genes in the adipose tissue. As the results showed, the mRNA levels of the target genes of PPARγ we tested were significantly decreased in PPARγ^3RA/+^ mice compared to those in WT mice (**Figure 5J**). Rosiglitazone treatment significantly induced the expression PPARγ target genes in WT mice; in PPARγ^3RA/+^ mice, rosiglitazone treatment restored the expression level of PPARγ target genes in PPARγ^3RA/+^ mice to levels similar to those of vehicle-treated WT mice (**Figure 5J**). Taken the rosiglitazone-induced metabolic improvement and gene expression restoration together, our results suggest that increasing PPARγ transcriptional activity could overcome the HFD-induced obesity and adipocyte hypertrophy in PPARγ^3RA/+^ mice.

## Discussion

In this study, based on the structural analysis of PPARγ/PPRE complex (Chandra et al., 2008), the roles of arginine at 134, 135, and 138 residues of PPARγ in binding PPRE, ligand (rosiglitazone) and cofactors (SRC1, SRC2, and NCoR) were verified by *in vitro* biochemical AlphaScreen and cell-based reporter assays. PPARγ may also possess regulatory mechanisms independent of DBD, such as PPARγ Ser273 phosphorylation mediated by CDK5 (Choi et al., 2011). Because previous reports have demonstrated that the regulation of PPARγ Ser273 phosphorylation can be detected by *in vitro* kinase assay in a reaction system including PPARγ LBD, CDK5, and ATP (Choi et al., 2011; Zheng et al., 2013), and the results from *in vitro* assay are consistent with that from *in vivo* assay, these results suggest that the post-translational phosphorylation of PPARγ at Ser273 by CDK5 is not rely on the existence of PPARγ DBD. Thus, the phosphorylation mediated by CDK5 may also be preserved in the 3RA mutant, although this has not been confirmed experimentally. Together, these data suggest that DBD-independent PPARγ regulations are intact in the PPARγ 3RA mutant. Therefore, we created a knock-in mouse model containing the PPARγ 3RA mutations. Homozygous PPARγ^3RA/3RA^ leads to embryonic death, suggesting the necessary role of the transcriptional activity of PPARγ for development. Chow diet fed PPARγ^3RA/+^ mice showed decreased transcriptional activity of PPARγ, while maintained comparable phenotypes with the WT littermate mice, suggesting that the one PPARγ alleles is sufficient to maintain the organismal metabolic network without stimuli. However, due to impaired transcriptional activity, PPARγ^3RA/+^ mice cannot sustain the burden of HFD stimuli, and appeared more severe insulin resistance and obesity. Accordingly, PPARγ agonist treatment rescued the activity of PPARγ, and restored the metabolic disorders in HFD-fed PPARγ^3RA/+^ mice. These results would indicate the important role of the transcriptional activity of PPARγ in protecting mice from HFD-stimulated metabolic disorders.

PPARγ plays crucial roles in maintaining the homeostasis of glucose and lipid metabolism. Over activating or debilitating its downstream signaling may cause the imbalance of the homeostasis (Rubenstrunk et al., 2007). For example, a water/glycerol transporting protein AQP7 regulates adipocyte glycerol efflux and influences lipid and glucose homeostasis. The deletion of AQP7 gene in mice leads to obesity and T2D (Rodriguez et al., 2006). However, it has also been reported that either increased or decreased AQP7 expression may lead to impaired glycerol dynamics and adipocyte hypertrophy (Oikonomou et al., 2018). Another example is that GLUT-4 is necessary for the insulin-regulated glucose uptake into muscle and fat cells which keeps the glucose homeostasis (Wu et al., 1998). However, if GLUT4 is over-expressed, it will send excess glucose into adipose tissue, leading to increased adipose cell hypertrophy and obesity (Shepherd et al., 1993). Also, overexpression of SCD1 in humans may be involved in the development of hypertriglyceridemia, atherosclerosis, and diabetes (Mar-Heyming et al., 2008). While inhibiting SCD1 function may also result in the accumulation of fatty acid metabolites that are deleterious to insulin signaling, and accordingly, the development of fatty acid-induced insulin resistance (Pinnamaneni et al., 2006). Thus, disorders of these genes will result in an imbalance of nutrients distribution and lead to obesity and diabetes. Rosiglitazone induces the expression of PPARγ target genes, which may provide a potential lighthouse to explain the adverse effects of long-term administration of TZD drugs (Rubenstrunk et al., 2007), as well as the metabolic improvement in HFD-fed PPARγ^3RA/+^ mice.

A number of laboratories have reported metabolic changes observed in heterozygous PPARγ-deficient mice (Barak et al., 1999; Kubota et al., 1999; Miles et al., 2000). Contrary to our finding, Kubota and colleagues reported that heterozygous PPARγ-deficient mice were protected from the development of insulin resistance caused by adipocyte hypertrophy after HFD-feeding. After administration of pioglitazone, the mice showed worsened phenotypes. Similarly, another group has reported improved insulin-sensitivity in an independently generated heterozygous PPARγ-deficient mouse model (Miles et al., 2000). These reports seem to approbate the negative roles of PPARγ transcriptional activity in metabolic regulation. However, PPARγ is required for adipose tissue development. Barak et al. found that the absence of PPARγ in mice leads to complete lipodystrophy (Barak et al., 1999), indicating the necessary role of PPARγ in lipid metabolism. It should be noted that besides transcriptional regulation, all of the five domains of PPARγ are involved in modulating the PPARγ signaling cascades (Wang S. B. et al., 2016). In this process, except transcriptional regulation by binding to PPREs, cofactors binding, and post-translational modifications including phosphorylation, acetylation, sumoylation, and ubiquitination throughout the full length of PPARγ also contribute to the functions regulated by PPARγ (Jin and Li, 2010; Ahmadian et al., 2013). However, both of the heterozygeous PPARγ-deficient mouse models by groups of Kubota and Barak eliminate most of the domains of PPARγ from DBD to the C-terminus (Barak et al., 1999), and thus the PPARγ in these models lost not only the transcription activity, but also other functional regulations. On the contrary, our PPARγ 3RA model is only mutated at three residues in DBD which contribute to PPARγ transcription deficiency, therefore may represent a suitable tool for the research of the role of transcriptional function of PPARγ in metabolism. The different phenotypes between PPARγ-deficient mice and PPARγ 3RA mutant mice further suggest that PPARγ needs to coordinate its transcriptional activity and its non-transcriptional regulatory actions for metabolic regulation.

Among the domains in nuclear receptors, the sequence of DBD shows the highest evolutionary conservation (Jin and Li, 2010; Helsen et al., 2012). Importantly, the three arginine residues we selected for mutation are conserved from birds to mammals including rodents and humans (**Supplementary Figure 4**), suggesting the conserved function or the PPARγ 3RA mutant in evolution, including humans. Additionally, the PPRE for PPARγ binding is also conserved with a direct repeats of hexameric sequence AGGTCA in different target genes, although each gene has distinct flanking sequence for its selective regulation (Khorasanizadeh and Rastinejad, 2001). Therefore, our PPARγ 3RA mutant model is a suitable tool for the research of PPARγ transcription in evolution.

In conclusion, we provide an alternative mouse model for further research on the transcriptional activity of PPARγ, and also for the drug discovery by targeting PPARγ. It should be noted that more detailed investigation about the DBD-independent PPARγ actions will further improve the significance of this mouse model. Considering the embryonic death of the PPARγ-3RA mice, future research will focus on creating homozygous conditional knockout mouse model with tissue specific PPARγ 3RA mutations to completely investigate the role of PPARγ transcriptional activity in specific tissues, particularly the adipose tissues and liver.

## Data Availability Statement

The raw data supporting the conclusions of this article will be made available by the authors, without undue reservation, to any qualified researcher.

## Ethics Statement

The animal study was reviewed and approved by the Laboratory Animal Center, Xiamen University.

## Author Contributions

YLi, LJ and YH designed the experiment, LJ and FG wrote and revised the manuscript. FG, SX, and YZ performed experiments. YLi and JT discussed and revised the manuscript. XZ assisted with the mice experiments. YLu and FG contributed to the structural analysis. All authors contributed to the article and approved the submitted version.

## Funding

work was supported by grants from the National Natural Science Foundation of China (81773793 and 31770814), the Fundamental Research Funds for the Central Universities (20720150052), the Programme of Introducing Talents of Discipline to Universities (B12001), and the National Science Foundation of China for Fostering Talents in Basic Research (J1310027).

## Conflict of Interest

The authors declare that the research was conducted in the absence of any commercial or financial relationships that could be construed as a potential conflict of interest.
