# Supplementary material for: PPARγ Transcription Deficiency Exacerbates High-Fat Diet-Induced Adipocyte Hypertrophy and Insulin Resistance in Mice
Source: Front Pharmacol. 2020 Aug 19;11:1285. doi: 10.3389/fphar.2020.01285 (PMC7466717; doi:10.3389/fphar.2020.01285)
Supplement: Supplementary file 1 [file DataSheet_1.pdf]

Supplementary Figure 1

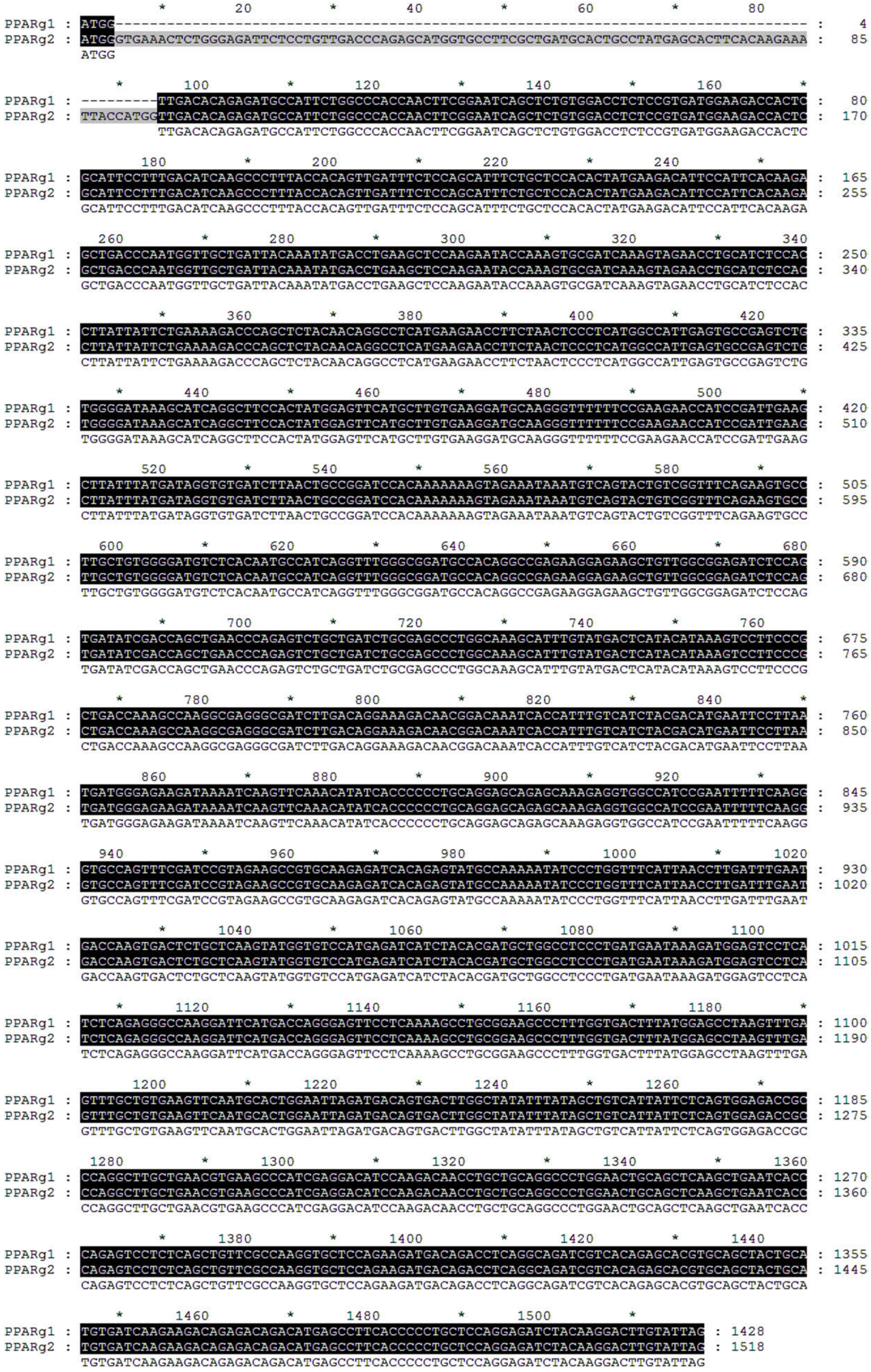

Supplementary Figure 1. The alignment of the CDS sequences of PPARγ1 (Genbank# NM_001127330.2) and PPARγ2 (Genbank# NM_011146.3).

Supplementary Figure 2

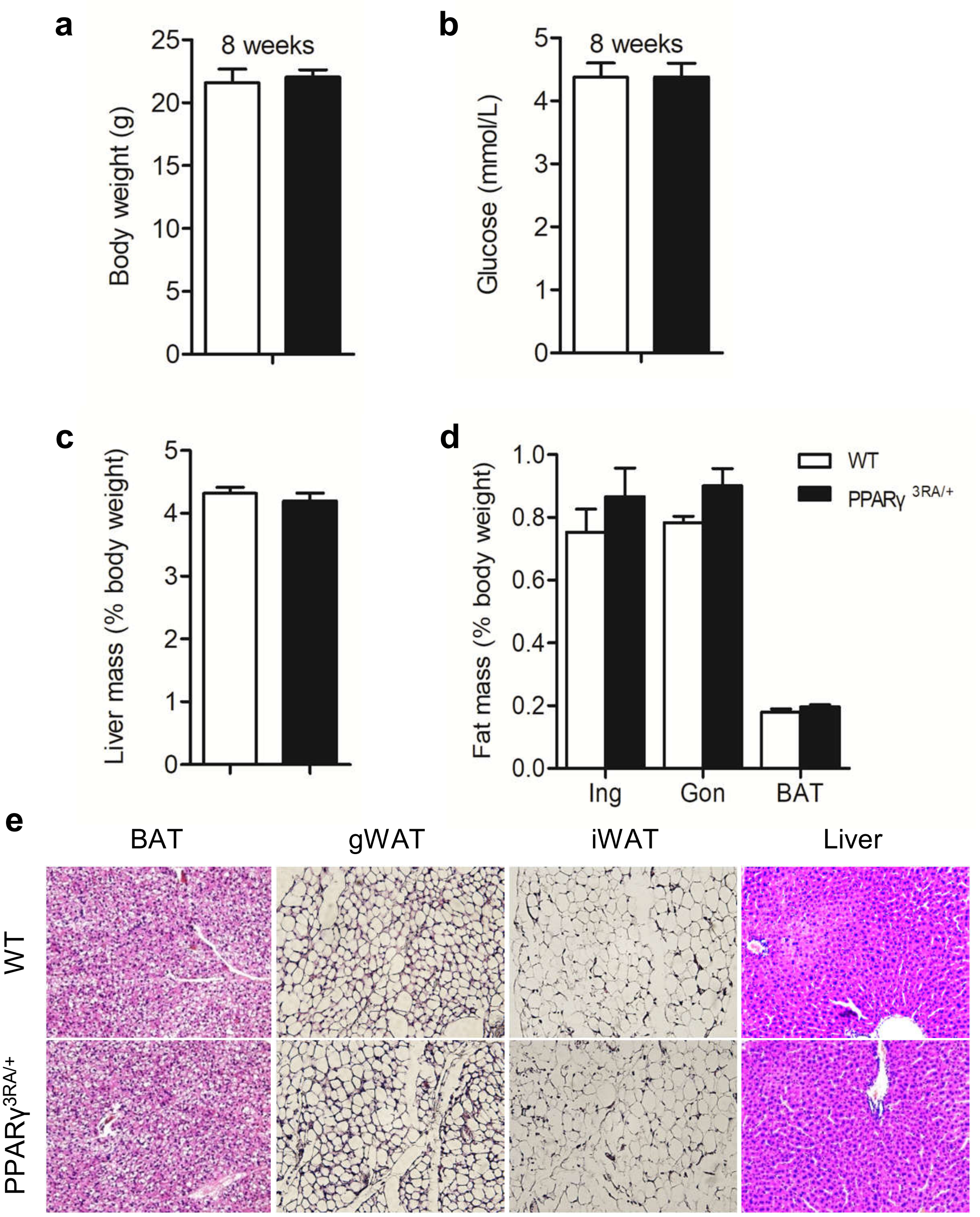

Supplementary Figure 2. Phenotype of PPARγ$^{3RA/+}$ and wild type (WT) littermates under chow diet. Male mice were fed with normal chow diet to 8 weeks old. (a) Body weight. (b) Fasting blood glucose level. (c) Liver/body weight ratio. (d) Fat/body weight ratio. (e) Representative H&E staining of the brown adipose tissue (BAT), gonadal white fat (gWAT), inguinal white fat (iWAT) and liver sections. n=12 per group. Students t-test. A P-value less than or equal to 0.05 is considered statistically significant.

Supplementary Figure 3

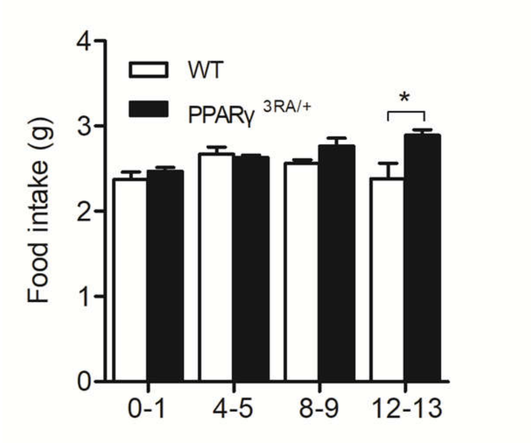

Supplementary Figure 3. Food intake of mice. After fed with chow diet to 8-week old, PPARγ$^{3RA/+}$ and wild type (WT) littermates were put to HFD for 15 weeks. The figure showed the HFD food intake of mice at different time course. n=12 per group. Students t-test. *P<0.05.

Supplementary Figure 4

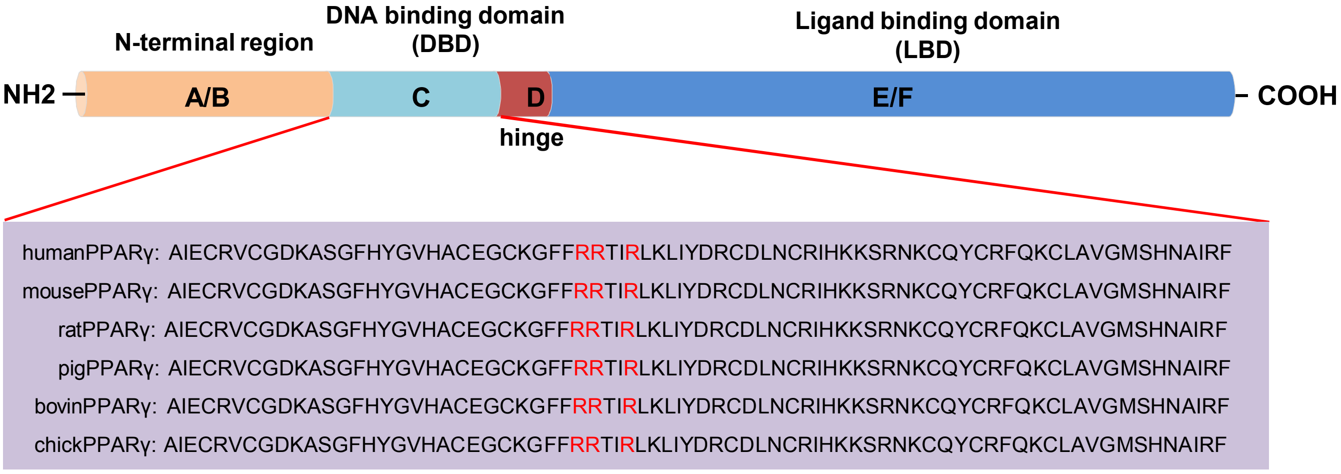

Supplementary Figure 4. Alignment of PPARγ DBD sequence in different species. Domains of nuclear receptors were shown. The mutated three arginine (R) residues were highlighted in red.

Supplementary Table 1. Sequences of the primers for qPCR.

Genes	Forward primer (5'-3')	Reverse primer (5'-3')
β-actin	TACAGCTTCACCACCACAGC	TCTCCAGGGAGGAAGAGGAT
PEPCK	CTCACTGACTCGGCTTACG	CCACTGAATGCAGACACTT
AQP7	TCGGTGTCAACTTGGGTTTTG	CCAGGTCATTCGGCCTAGTG
AP2	AAGGTGAAGAGCATCATAACCCT	TCACGCCTTTCATAACACATTCC
SCD1	ACCTGCCTCTTCGGGATTTT	GTCGGCGTGTGTTTCTGAGA
LPL	TGAAAGTGGGTTTTCCTGAGTAT	GGTTAGCCACCGTTTAATATTTG
GLUT4	CCTTCTATTTGCCGTCCTCC	TGGCCCTAAGTATTCAAGTTCTG
CPT1b	TCTTCTTCCGACAAACCCTGA	GAGACGGACACAGATAGCCC
CD36	CCTCTTCCCGTCAGACTTGTG	TCGGAGGTGTTGGCTAGTATTT
